# Comment on Pietrapertosa et al. How to Prioritize Energy Efficiency Intervention in Municipal Public Buildings to Decrease CO_2_ Emissions? A Case Study from Italy. *Int. J. Environ. Res. Public Health* 2020, *17*, 4434

**DOI:** 10.3390/ijerph18083961

**Published:** 2021-04-09

**Authors:** Miroslav Variny

**Affiliations:** Department of Chemical and Biochemical Engineering, Faculty of Chemical and Food Technology, Slovak University of Technology in Bratislava, Radlinského 9, 812 37 Bratislava, Slovakia; miroslav.variny@stuba.sk

**Keywords:** heat losses, energy saving measures, lighting refit, investment prioritization, CO_2_ emissions reduction

## Abstract

This paper responds to the article by Pietrapertosa et al., doi:10.3390/ijerph17124434, published previously in the International Journal of Environmental Research and Public Health. Its aim is to discuss the appropriateness of the studied method, to analyze its weak sides and to propose its robustness improvement. Thus, data presented in the above study were examined and recalculated, yielding, among others, indicators of annual energy savings (in kWh per m^2^ of total heated area) and specific proposals investment costs (in € per m^2^ of total heated area). By analyzing the obtained data for all public buildings, a significantly simplified approach to this problematic has been suggested while several other features of the research method and some presented results lack proper reasoning and discussion. Individual approach to each public building has been proposed and discussed point-by-point to enhance the method’s applicability. As a result, more realistic outcomes are obtained, and suitable investment actions can be proposed.

## 1. Introduction

The paper by Pietrapertosa et al. [1] presents a method and obtained results in energy saving proposals in public buildings in Potenza Municipality. The problem of energy consumption decrease in public and residential buildings resonates in the European society for decades [2,3]. Step by step legislation changes and development of new materials and technologies support deep renovation activities [4] such as outer envelope insulation, windows replacement, air conditioning system installation, photovoltaic panels installation, heat source (boiler) replacement, lighting system refit, etc. EU require all buildings constructed in 2020 and later to meet the conditions of nearly-zero energy buildings (nZEB) and support the renovation activities in older ones to reach or to approach this energy standard [5]. Given this legislation frame, public buildings refit aimed at a substantial cut in electric energy and heat consumption is the right choice. However, reasonable means of energy savings estimation and prioritization of the resulting investments must be based on a robust method. This study analyzes the paper by Pietrapertosa et al. [1] in terms of method robustness and obtained results feasibility, discusses the method’s weak points, and proposes its improvement.

## 2. General Comments

As input values for carbon dioxide emissions reduction due to fuel (natural gas) and electricity saving proposals, annual natural gas (NG) consumption of 10.8 GWh (higher heating value) and annual electricity consumption of 10.9 GWh were considered in the case study with the corresponding CO_2_ emissions amounting to 4428 and 4469 t, respectively. As a reference, year 2016 is mentioned but no information on the source of emission factors is provided. By simple recalculation, almost identical emission factor of around 400 g CO_2_/kWh was obtained both for natural gas and electricity. Considering a typical higher heating value of European NG of 54 GJ/t, slightly simplified composition of 99% wt. of methane and 1% wt. of inert gas, methane combustion stoichiometry and CO_2_ to methane molar mass ratio of 2.75, results in a more realistic NG emission factor of around 180 g of CO_2_ per kWh higher heating value, or of around 200 g of CO_2_ per kWh lower heating value. This complies with the NG emission factor applied by D´Amico et al. [6].

Average electricity emission factors differ for each country based on its energy mix. To obtain as realistic model input data as possible, the authors should use recent emission factor and refer to the data source. Moreover, the average electricity emission factor might not be representative enough if considering electricity saving measures in public buildings (photovoltaic panel installation) that are active during the day only. The share of individual electricity sources varies in the energy mix during the day and so does the best way to assess this is marginal emission factor [7] as it can differ by tens of percent from the average one.

Final aim of energy saving measures is not defined in terms of primary energy consumption reduction. What building energy class is to be achieved by the proposed retrofit? Should nZEB conditions be achieved [8,9]? Performing a public building retrofit means that the proposal of energy saving measures cannot just follow the operational costs reduction but has to meet legislation criteria imposed on the building energy class after the retrofit [10,11]. In addition, information on the current building energy class would be useful to relate the expected energy savings.

Energy consumption reduction proposals considered in paper [1] should be discussed in more detail:

- Installation of a ventilation system to achieve natural gas savings. We can thus assume that there is no air conditioning system installed in the buildings. If this holds true, a new air conditioning unit will certainly provide thermal energy consumption reduction but, on the other hand, it will increase electricity consumption [12]. This fact is completely omitted in the analyzed study.
- Installation of a “more efficient” cooling system indicates that cooling systems are already present in all buildings (which is improbable but possible) and should be refitted. In such a case, why should a “more efficient” cooling system adoption increase electric energy consumption as presented in the study.
- Lighting refit is proposed. Given a probably wide range of existing lighting system quality, reliability, age, etc., such measure cannot be evaluated without a site visit [13]. Taking the building of our faculty as an example: the lighting is partly refitted, partly dysfunctional and there is no relevant information on how its operation time relates to working hours. Similar situation might be expected in the analyzed buildings, the range of factors influencing the final proposal being extended by different years of building construction. Moreover, lighting refit does not mean the replacement of light sources only. Rewiring and switch rooms adjustment might be required, especially in older buildings, which increases the investment cost significantly. On the other hand, if the existing lighting system is outdated, it can break down frequently and its repairs can be costly. All this (and possibly more factors) must be considered in a sound lighting refit proposal.
- Installation of photovoltaic panels (PV) is considered in all buildings. A brief dedicated discussion about their sizing is required. Should they cover the building’s own consumption predominantly, or is an extensive electricity surplus expected, sold to the local grid for a certain feed-in tariff? Why is building no. 3 to be equipped with far more panels than any other building? Of course, it is the largest one considered but it has the shortest annual working hours, so the produced electricity will be mostly exported to the grid. Moreover, based on data in [1] it can be calculated that total annual PV electricity production is the same for office buildings and school buildings. Is it just a random surprising result or was it designed on purpose?

## 3. Data Analysis Method

The study contains various relevant information regarding the expected annual energy savings and the total investment costs. The data listed in [1] underwent recalculation using the following indicators: savings ratio (*SR*); specific annual heat saving (*SHS*); specific electricity consumption savings (*SS_E_*); specific investment (*SI*); return on investment (*ROI*); and specific cost of CO_2_ emissions reduction (*SC_CO2_*). Moreover, the role of building geometry and disposition is examined regarding heat losses through outer walls and roof.

Savings ratio, *SR* (kWh/kWh), expresses the ratio of anticipated annual energy savings due to outer walls insulation to that due to other (*i-th*) heat saving measures considered (windows replacement, roof insulation, new air conditioning), Equation (1).

(1) $$SR_{ow,i,j}=\frac{AS_{ow,j}}{AS_{i,j}},\;i=wi,r,ac$$

where *AS* stands for annual savings (kWh/year), *j* is the building number (1 to 25), *wi* represents windows replacement, *r* roof insulation, ow outer walls insulation, and *ac* means air conditioning unit refit.

Specific annual heat savings, *SHS_ow_* (kWh/m^2^), represent intensity reduction of heat losses through outer walls due to their insulation. It is calculated by Equation (2), where *A_h_* (m^2^) is the total heated area of the *j*-*th* building. It is a commonly evaluated measure in building insulation [14,15].

(2) $$SHS_{ow,j}=\frac{AS_{ow,j}}{A_{h,j}}$$

Specific electricity consumption savings, *SS_E_* (W/m^2^), due to lighting refit is defined by Equation (3) and provides insight into the related electricity consumption efficiency increase. $AS_{l,j}$ stands for annual electricity consumption reduction by lighting refit in the *j*-*th* building and *WH_j_* denotes annual working hours in this building.

(3) $$SS_{E,j}=\frac{AS_{l,j}}{A_{h,j}.WH_{j}}$$

Specific investment, *SI* (€/m^2^), enables comparing individual energy saving measures in terms of their economic requirements, Equation (4), with $I_{i,j}$ (€) standing for investment cost of the *i*-*th* energy saving measure in the *j*-*th* building.

(4) $$SI_{i,j}=\frac{I_{i,j}}{A_{h,j}}$$

Return on investment, *ROI* (years), for the *i*-*th* energy saving measure in the *j*-*th* building is evaluated by Equation (5) as the ratio of investment cost and annual financial benefit. It is calculated as the product of the expected annual savings and the unit cost of saved energy, *c_en_* (€/kWh), with *en* denoting either natural gas (NG) or electricity (E).

(5) $$ROI_{i,j}=\frac{I_{i,j}}{AS_{i,j}.c_{en}}$$

Specific cost of CO_2_ emissions reduction, *SC_CO2_* (€/t), relates the economics and the associated environmental benefit of applying individual measures. It is calculated by Equation (6), where an average 25-year lifetime period of energy saving measures is assumed and emission factors *EF_en_* of 180 kg/kWh for natural gas and 400 kg/kWh for electricity are adopted.

(6) $$SC_{CO2,i,j}=\frac{I_{i,j}}{AS_{i,j}.EF_{en}.25}$$

Values of *A_h,j_*, *AS_i,j_*, *WH_j_*, *I_i,j_*, and *n_j_* were extracted from data Table A1, Table A2 and Table A3 in Appendix A.

Savings ratio, $SR_{ow,r}$, calculated by Equation (1) deserves further attention. It is expected to vary both with actual technical state of the building as well as with its size and geometric characteristics and the considered insulation properties and thickness.

As for the impact of the size and the geometric characteristics, the ratio of outer walls area to roof area can be expressed for a tetrahedral building with a rectangular basis as follows, Equation (7):

(7) $$AR=\frac{A_{ow}}{A_{r}}=\frac{2\left(a+b\right)hn}{ab}$$

for different ratios *k* = *b*/*a* it can be rewritten as Equation (8):

(8) $$AR=\frac{2\left(a+kb\right)hn}{aka}=\frac{2\left(k+1\right)}{k}.\frac{hn}{a}$$

So, obviously, the value of the ratio of outer walls area to roof area, *AR*, decreases as “*a*” increases, which means that for buildings with the same number of floors and the same floor height but with increasing size, the roof gradually gains importance as a heat exchange surface compared to building walls. The values of *AR* for various arrangements are calculated in Table 1 and Table 2.

Data presented in Table 1 and Table 2 show quite significant AR value differences for buildings with dimensions like those in study [1]. For a single floor building with the roof area of around 1000 m^2^ (*j* = 25) it is in the range of 0.5 to 0.6, while for a three-story building with the roof area of around 400 m^2^ (*j* = 21) it exceeds 2.4. The ratio of heat losses through outer walls and through the roof varies in the same range if the same heat resistance is assumed. Thus, the $SR_{ow,r}$ values should also show this trend if similar percentual heat losses reduction by insulation is assumed.

## 4. Results and Discussion

As a first step, ratios of annual energy savings due to outer walls insulation to annual energy savings due to other measures (*SR_ow,i,j_*) were evaluated and the results are shown in Table 3. Moreover, annual heat consumption reduction per m^2^ of heated area (*SHS_ow,j_*) and annual electric energy consumption reduction due to lighting refit per m^2^ of heated area (*SS_E,j_*) were evaluated and are presented in Table 3 as well.

Due to the wide range of possible building technical states, their various construction years ranging from the end of the 19th to the end of the 20th century and the resulting development of construction materials quality and technical norms in construction [16,17], various *SR_ow,i,j_* values are expected for individual buildings. Contrary to this expectation, identical values for several buildings can be seen in Table 3. As an example, all single floor buildings have the same *SR_ow,i,j_* values regardless of their total heated area or year of construction. Similarly, two-story and three-story buildings have the same *SR_ow,wi,j_* values. The same holds true for the *SR_ow,r,j_* values and similar trends can be seen for the *SR_ow,ac,j_* values. This documents that the chosen energy saving calculation method does neither consider the inevitably existing differences in heat losses through outer walls, windows, and roofs of individual buildings, nor their economically acceptable reduction rate. Moreover, the *SR_ow,r,j_* values do not reflect the influence of buildings geometry or their sizes, both of which are relevant factors as documented in Table 1 and Table 2 and the related discussion. Such calculation method can be applied to very preliminary estimations only in the absence of construction and field data and should be considered with caution. Far more data must be acquired, including site visit results, to perform reliable calculations and to obtain relevant inputs for economic analysis and subsequent recommendations of investment priorities. A more complex approach, such as that used by Salvalai et al. [18] is recommended. 

Similarly to *SR_ow,i,j_* values, *SS_E,j_* values are almost identical, regardless of the building. This must be considered as a significant simplification and is irrelevant with respect to decision making on building energy consumption reduction measures.

The only truly varying parameter in Table 3 is the annual heat consumption reduction due to outer walls insulation per m^2^ of total heated area. Its range of 15 to over 127 kWh/m^2^ is quite wide. Energy consumption reduction of around or below 20 kWh/m^2^ hardly suffices to improve the building energy class by one grade. On the other hand, values of or above 60 kWh/m^2^ indicate quite extensive heat losses in the current state. As stated in Zinzi et al. [12], the average annual heat consumption in an Italian school is around 130 kWh/m^2^ which does not contradict the expected annual savings of several tens of kWh/m^2^.

Comparison between buildings no. 5, 6, and 23 yields surprising results. All of them are single floor buildings with similar total heated area and with the year of construction difference of a few years only. However, outer walls insulation yields heat savings of 100 kWh/m^2^ in building no. 23 but only 14.5 kWh/m^2^ in building no. 5. In contrast to this are the three-story buildings no. 20 and 21, with similar total heated areas, both exhibiting the *SHS_ow_* value of around 55 kWh/m^2^ but with the construction year difference of almost 100 years. An alternative explanation is a typing error in [1], with the true construction year of building no. 20 being 1988 instead of 1888.

It can be concluded that the values of expected heat consumption reduction due to individual saving measures deserve more attention and should be discussed more closely in the study by Prietrapertosa et al. [1]. Moreover, recalculation of results shown in Table 3 indicates that heat savings due to other measures analyzed were considered directly proportional to the expected heat savings by outer walls insulation, which is an oversimplification. Similarly, the expected electric energy savings due to lighting refit are evidently a rough estimate due to the lack of actual design and operation data [19] and findings acquirable by site visit only [13,20].

It should be also noted that outer envelope and roof insulation as well as windows replacement contribute to increased airtightness of the buildings [21,22]. This must be compensated by increased ventilation rate, e.g., by more intense air conditioning unit operation with an associated increase in electricity consumption. 

Like expected annual heat and electricity savings, the corresponding investment costs were also analyzed closely. Calculated specific investment costs of individual saving measures in individual buildings (*SI_i,j_*) are presented in Table 4. Specific costs of windows replacement (*SI_wi,j_*) follow a reasonable trend, being the lowest in single floor buildings where the associated costs are lower than in other buildings. The highest costs were expected in office buildings which, again, is understandable due to the number of floors (3 to 5) and probably untypical window design. Specific costs of roof insulation (*SI_r,j_*) on the other hand are almost the same for single floor and three-story buildings which is questionable given the varying roof to total heated area ratio. Specific costs of air condition system replacement (*SI_ac,j_*) do not follow any specific trend. Specific costs of outer walls insulation (*SI_ow,j_*) are almost the same for single floor, two-, and three-story buildings. This appears to be a simplification given the additional costs expected in multi-story buildings (renting scaffolds, material transport etc.). Last, a single value of 18.8 €/m^2^ of lighting refit cost was obtained, again indicating a significant simplification, and decreasing the relevance of investment costs estimation towards decision making on future investments. The actual technical state of the lighting system, wiring and many other factors have to be considered if a reasonable estimate of the lighting system refit is to be obtained.

Return on investment (*ROI*), and specific cost of CO_2_ emissions reduction (*SC_CO2_*) were calculated based on the data from Table A1, Table A2 and Table A3 in Appendix A and are shown in Table 5. A general survey of the obtained *ROI* values yielded that lighting system refit and outer walls and roof insulation generally exhibit the shortest *ROI* values while the *ROI* values for air conditioning system replacement mostly exceed 15 years and those for windows replacement are even higher. This is reflected also in the SC_CO2_ values, where lighting refit performs similarly to outer walls and roof insulation showing values of around 100 €/t. This means that if carbon tax of around 100 €/t is assumed, investment costs of these savings proposals could be almost completely covered by the decreased CO_2_ emissions. Such proposals can then be considered environmentally feasible. The remaining saving proposals showed worse results.

Considering the findings discussed above, the outer walls insulation recommended in [1] is hardly “the best energy efficiency intervention for all the considered buildings” if lighting refit offers comparable benefits and environmental performance. The authors did not offer any supplementary explanation. Similarly, no explanation is provided why the lighting system refit in some schools is marked with green color (most favorable energy saving option) in Table 2 in [1], in some schools with light green and in other ones with yellow (less favorable energy saving option), if it offers the same economic and environmental parameters in all schools. Roof insulation in building no. 3 is marked as a quite favorable option (light green) while it exhibits a *ROI* of over 30 years. Several other examples of questionable energy saving measures favorizing can be found. 

Interesting information were obtained by comparing data on overall annual heat and electricity consumption in office buildings and school buildings with the achieved annual heat and electricity savings according to [1]. Office buildings (nos. 1 to 4) consume 1,000,000 kWh of heat and 1,300,000 kWh of electricity annually, while school buildings (nos. 5 to 25) have an overall annual heat consumption of 4,800,000 kWh and overall annual electricity consumption of 850,000 kWh. Total calculated annual heat savings due to outer walls and roof insulation, windows replacement, and air conditioning system refit amount to 546,153 kWh in office buildings and to 2,609,908 kWh in school buildings. Similarly, the sum of annual electricity consumption savings due to lighting refit and annually produced electric energy due to PV panels’ installation is 394,500 kWh in office buildings and 604,313 kWh in school buildings. This corresponds to the achieved relative consumption reduction of 54.5% for heat and of 46.5% for electric energy in both building types. Given the large variability and differences in school and office building typologies, years of construction, actual technical state and many more factors influencing the consumption of heat and electricity as well as their reduction, this seems improbable. As a result, further discussion requiring additional data on the actual situation and proposed energy saving measures, not provided in [1], is necessary.

Furthermore, several comments can be made to data presented in Table 4 in [1]. The authors recommended dividing the selected energy saving measures into three realization phases. Annual energy savings and CO_2_ emission savings for individual phases are as follows: phase 1: 508,812 kWh and 191.31 t; phase 2: 492,570 kWh and 278.45 t; and phase 3: 1,048,730 kWh and 173.80 t. Phase 1 comprises lighting refit only (electric energy saving), phase 2 includes PV installation and heat source replacement (combined electric energy and heat), and phase 3 contains outer walls and roof insulation (heat savings). So, emission factor of electric energy and natural gas should be obtained from these data for phases 1 and 3, respectively. Emission factors calculated from the data above are as follows: phase 1: 0.376 t_CO2_/kWh; phase 2: 0.565 t_CO2_/kWh; and phase 3: phase 0.166 t_CO2_/kWh. Obviously, none of these values fits the emission factor of around 0.4 t_CO2_/kWh considered both for electricity and natural gas in [1]. That for the phase 3 is even lower than the real NG emission factor of around 0.18 t_CO2_/kWh. Emission factor for phase 2 is higher than that for electricity savings, which is infeasible. No additional explanation is provided in [1]. A possible explanation is that the new heat source installation in two school buildings relates to fuel switch from liquefied petroleum gas (LPG) to NG. Thus, CO_2_ emissions savings result both from fuel saving as well as from using fuel with lower emission factor (0.18 t_CO2_/kWh for NG compared to around 0.21 t_CO2_/kWh for propane and around 0.23 t_CO2_/kWh for butane). No information on the different emission factors for LPG and NG is provided in [1]. It can thus be concluded that the presented data regarding CO_2_ emissions savings in individual phases are questionable and should be checked.

## 5. Recommendations for Research Method Improvement

- The above analysis and results identified several weak spots in the method used in [1] to propose, evaluate and prioritize energy saving actions in public buildings. The following key issues should be incorporated to make the method more robust:
- Definition of a clear objective of the energy saving measures should be stated first. nZEB conditions should be referred to as a sort of golden standard and reasons why these conditions cannot be met in individual buildings should be explained. This is the very first step that determines which energy saving measures should be considered in which building.
- Specificities of individual public buildings should be considered in more detail. Deeper operation and design data analysis together with dedicated site visit provide important data. Omitting this research step yields quite simplified results which are unsuitable as a basis for decision making. Feasibility of lighting retrofit proposal is exceptionally dependent on reliable input data, which can be obtained by site visit only, coupled with dedicated measurements.
- PV installation has to be considered with care. An analysis of the current situation and an outlook on the expected feed-in tariffs for electricity surplus should be a part of this analysis.
- Environmental assessment of proposals should work with feasible emission factors. The use of marginal electricity emission factor is recommended whenever this is known. Heat source replacement for a more efficient one, including fuel switch, should consider different emission factors for individual fuels.

## 6. Conclusions

A thorough analysis of the study by Pietrapertosa et al. [1] was conducted yielding several findings specific to the case study as well as some of general nature. Several weak spots in the research method have been identified and recommendations on its robustness improvement were formulated. Most important issues included: lack of clear method objectives statement and formalized approach omitting deeper design and operation data analysis or inputs from site visits. In addition, discrepancies in the reported CO_2_ savings were found and the obtained overall heat and electricity consumption reduction percentages in office buildings and school buildings are worth further debate. This commentary paper will hopefully aid the scientific debate on energy saving proposals in public buildings and help the authors to better focus their future research in this field.

## Figures and Tables

**Table 1:** Values of the ratio of outer walls area to roof area, *AR*, according to Equation (8) with typical value of h = 4 m for a single floor building (*n* = 1).

$A_{r}\;(\mathrm{Is}\;\mathrm{Equal}\;\mathrm{to}\;A_{h}),\;m^{2}$	k = 1 (Square Basis)	k = 2 (Typical Rectangular Basis)	k = 4 (Long Narrow Rectangular Basis)
200	1.13	1.20	1.41
400	0.80	0.85	1.00
600	0.65	0.69	0.82
800	0.57	0.60	0.71
1000	0.51	0.54	0.63
1500	0.41	0.44	0.52

**Table 2:** Values of *AR* according to Equation (8) with typical value of h = 4 m for a three-story building (*n* = 3).

$A_{r}\;(\mathrm{Is}\;\mathrm{Equal}\;\mathrm{to}\;A_{h}),\;m^{2}$	k = 1 (Square Basis)	k = 2 (Typical Rectangular Basis)	k = 4 (Long Narrow Rectangular Basis)
200	3.39	3.60	4.24
400	2.40	2.55	3.00
600	1.96	2.08	2.45
800	1.70	1.80	2.12
1000	1.52	1.61	1.90
1500	1.24	1.31	1.55

**Table 3:** Relative energy performance recalculation for individual energy saving measures. *SR* = savings ratio (kWh/kWh), *SHS* = specific annual heat savings (kWh/m^2^), *SS_E_* = specific electricity consumption reduction due to lighting refit (W/m^2^). *j* = building number (*j* = 1 to 25), *ow* = outer walls insulation, *wi* = windows replacement, *r* = roof insulation, *ac* = air conditioning unit refit. Color code: yellow—single floor building, green—two-story building, light blue—three-story building, white—four- or five-story building.

***j***	$SR_{ow,wi,j}$	$SR_{ow,r,j}$	$SR_{ow,ac,j}$	$SHS_{ow,j}$	$SS_{E,j}$
1	5.92	3.90	31.27	54.38	7.76
2	7.33	4.92	32.95	67.57	7.77
3	18.62	3.90	31.26	27.30	6.18
4	3.51	3.90	31.26	36.35	6.18
5	5.71	6.50	13.03	14.44	6.41
6	5.69	6.48	13.03	73.75	6.41
7	4.41	4.94	12.37	65.05	6.41
8	5.70	6.50	13.03	60.82	6.41
9	4.29	4.94	8.66	47.19	6.41
10	5.69	6.52	12.16	71.46	6.41
11	4.34	4.94	10.18	54.10	6.41
12	4.41	4.94	12.37	99.98	6.41
13	4.41	4.94	12.37	37.59	6.41
14	5.71	6.50	13.03	71.08	6.41
15	4.29	4.94	8.66	127.66	6.41
16	4.37	4.95	10.83	42.93	6.41
17	5.45	6.50	8.10	32.58	6.41
18	4.37	4.94	10.83	30.83	6.41
19	4.29	4.94	8.67	29.29	6.40
20	4.29	4.94	8.66	56.36	6.41
21	4.41	4.94	12.38	54.44	6.41
22	4.41	4.94	12.37	61.48	6.41
23	5.65	6.51	11.40	100.09	6.41
24	4.40	4.94	12.38	95.25	6.41
25	5.70	6.49	13.03	102.49	6.41

**Table 4:** Relative economic performance recalculation for individual energy saving measures. *SI* = specific investment cost per m^2^ of total heated area (€/m^2^), l = lighting refit. Color code: yellow—single floor building, green—two-story building, light blue—three-story building, white—four- or five-story building.

***j***	$SI_{ow,j}$	$SI_{wi,j}$	$SI_{r,j}$	$SI_{ac,j}$	$SI_{l,j}$
1	64.2	92.6	14.3	5.1	18.8
2	74.6	77.4	12.0	14.9	
3	33.7	71.3	12.8	3.0	
4	34.5	71.3	9.6	5.2	
5	34.7	47.5	6.4	6.9	
6	32.3	47.6	6.4	6.5	
7	37.5	59.4	10.6	4.4	
8	41.3	47.5	6.4	4.1	
9	32.6	59.4	8.0	3.9	
10	35.1	47.4	6.4	7.0	
11	37.2	59.4	8.0	14.9	
12	37.0	59.4	8.0	7.9	
13	33.4	59.4	8.0	3.3	
14	35.0	47.5	6.4	5.2	
15	24.2	59.4	8.0	17.0	
16	28.8	59.3	8.0	5.8	
17	38.1	47.5	6.4	3.0	
18	26.0	59.4	8.0	10.4	
19	35.1	59.4	8.0	14.0	
20	31.8	59.4	8.0	5.3	
21	36.1	59.3	8.0	4.5	
22	37.0	59.4	8.0	6.9	
23	43.8	47.4	6.4	8.8	
24	37.0	59.4	8.0	9.3	
25	34.4	47.5	6.4	8.0	

**Table 5:** Reevaluation of basic economic and environmental parameters of individual energy saving measures. *ROI* = return on investment (years), *SC_CO2_* = specific cost of CO_2_ emissions reduction (€/t).

*j*	Outer Walls		Windows		Roof		Air Conditioning		Lighting	
	$ROI_{j}$	$SC_{CO2,j}$	$ROI_{j}$	$SC_{CO2,j}$	$ROI_{j}$	$SC_{CO2,j}$	$ROI_{j}$	$SC_{CO2,j}$	$ROI_{j}$	$SC_{CO2,j}$
1	19.8	262	169.4	2239	17.3	229	49.6	656	3.7	70
2	18.6	245	141.2	1867	14.7	194	122.3	1617	3.7	70
3	20.7	274	817.1	10,803	30.6	405	57.2	757	8.4	162
4	16.0	211	115.8	1531	17.2	228	74.9	990	8.4	162
5	40.4	534	315.5	4171	48.2	638	105.3	1392	7.3	139
6	7.4	97	61.8	817	9.4	125	19.2	254		
7	9.7	128	67.6	894	13.6	179	14.0	185		
8	11.4	151	74.9	990	11.5	151	14.9	196		
9	11.6	153	90.7	1199	14.0	185	12.1	159		
10	8.3	109	63.4	838	9.7	129	20.1	265		
11	11.6	153	80.1	1059	12.2	162	47.1	622		
12	6.2	82	44.0	581	6.6	87	16.4	216		
13	14.9	198	116.9	1546	17.6	233	18.5	244		
14	8.3	109	64.1	847	9.8	130	16.2	214		
15	3.2	42	33.5	443	5.2	69	19.3	256		
16	11.3	149	101.5	1342	15.4	204	24.4	323		
17	19.7	260	133.5	1765	21.4	283	12.7	169		
18	14.2	188	141.3	1869	21.5	284	61.5	813		
19	20.1	266	146.1	1931	22.6	299	69.9	924		
20	9.5	125	75.9	1004	11.7	155	13.7	181		
21	11.1	147	80.8	1068	12.2	161	17.2	228		
22	10.1	134	71.5	945	10.8	142	23.5	311		
23	7.4	97	45.0	595	7.0	92	16.8	222		
24	6.5	86	46.2	610	6.9	92	20.2	267		
25	5.6	75	44.5	588	6.8	90	17.2	227		

## Data Availability

All data obtained by calculations and analyses are listed directly in this study.

## Appendix A

**Table A1:** Basic characteristics of buildings. *A_h_* = total heated area (m^2^), *j* = building number (1 to 25), *n* = number of floors, WH = working hours per year [1].

*j*	Typology	*A_h_*, m^2^	*n*	Year of Construction	WH, Hours/Year
1	Office building	974	4	1903	3432
2		335	3	1925	3432
3		6305	5	1988	1872
4		3619	4	1975	1872
5	School, kindergarten	720	1	1981	2100
6		773	1	1984	
7		2000	2	1984	
8		1514	1	1959	
9		1920	3	1969	
10		712	1	1993	
11		336	2	1968	
12		2700	3	1942	
13		1496	2	1942	
14		1430	1	1983	
15		516	2	1968	
16		869	2	1977	
17		1640	1	1994	
18		480	2	1980	
19		356	3	1973	
20		1179	3	1888	
21		1385	3	1981	
22		2700	3	1975	
23		856	1	1975	
24		2027	2	1976	
25		1089	1	1981	

**Table A2:** Investment costs for individual buildings and energy saving measures considered. *I* = investment cost (€), *ow* = outer walls insulation, *wi* = windows replacement, *r* = roof insulation, *ac* = air conditioning unit refit, l = lighting refit, *j* = building number (1 to 25) [1].

*j*	*I_ow_*	*I_wi_*	*I_r_*	*I_ac_*	*I_l_*
	€				
1	62,500	90,155	13,961	5000	18,270
2	25,000	25,935	4016	5000	6285
3	212,500	449,350	80,410	18,750	118,219
4	125,000	257,925	34,616	18,750	67,862
5	25,000	34,200	4590	5000	13,500
6	25,000	36,813	4941	5000	14,494
7	75,000	118,750	21,250	8750	37,500
8	62,500	71,963	9659	6250	28,388
9	62,500	114,000	15,300	7500	36,000
10	25,000	33,725	4526	5000	13,350
11	12,500	19,950	2678	5000	6300
12	100,000	160,313	21,516	21,250	50,625
13	50,000	88,825	11,921	5000	28,050
14	50,000	67,925	9116	7500	26,813
15	12,500	30,638	4113	8750	9675
16	25,000	51,538	6918	5000	16,294
17	62,500	77,900	10,455	5000	30,750
18	12,500	28,500	3825	5000	9000
19	12,500	21,138	2838	5000	6666
20	37,500	70,063	9404	6250	22,106
21	50,000	82,175	11,029	6250	25,969
22	100,000	160,313	21,516	18,750	50,625
23	37,500	40,613	5451	7500	16,050
24	75,000	120,413	16,161	18,750	38,006
25	37,500	51,775	6949	8750	20,419

**Table A3:** Annual saving of energy in kWh/year for individual buildings and energy saving measures considered. *AS* = annual saving (kWh/year), *ow* = outer walls insulation, *wi* = windows replacement, *r* = roof insulation, *ac* = air conditioning unit refit, l = lighting refit, *j* = building number (1 to 25) [1].

*j*	*AS_ow_*	*AS_wi_*	*AS_r_*	*AS_ac_*	*AS_l_*
	kWh/Year				
1	52,964	8946	13,565	1694	25,954
2	22,636	3087	4599	687	8928
3	172,133	9243	44,145	5506	72,906
4	131,560	37,435	33,744	4209	41,851
5	10,395	1822	1599	798	9686
6	57,005	10,014	8793	4376	10,400
7	130,096	29,529	26,335	10,513	26,907
8	92,080	16,147	14,176	7069	20,369
9	90,606	21,126	18,341	10,460	25,831
10	50,877	8943	7805	4185	9579
11	18,177	4185	3682	1785	4520
12	269,943	61,272	54,644	21,815	36,324
13	56,239	12,767	11,386	4545	20,126
14	101,642	17,812	15,637	7803	19,238
15	65,871	15,365	13,340	7608	6942
16	37,310	8532	7540	3444	11,691
17	53,434	9809	8221	6593	22,064
18	14,796	3389	2995	1366	6458
19	10,427	2432	2112	1203	4783
20	66,445	15,505	13,462	7671	15,862
21	75,396	17,099	15,248	6092	18,633
22	166,003	37,680	33,604	13,415	36,324
23	85,675	15,166	13,165	7517	11,516
24	193,074	43,837	39,096	15,600	27,270
25	111,614	19,577	17,187	8569	14,651
